# The Dynamic Role of Liquid Biopsy in Patients with Non-Small Cell Lung Cancer Receiving Immune Checkpoint Inhibitors: Emerging Opportunities and Clinical Challenges

**DOI:** 10.3390/cancers18193240

**Published:** 2026-10-08

**Authors:** Tancredi Didier Bazan Russo, Valerio Gristina, Francesco Pepe, Claudia Scimone, Giulia Busuito, Domenico Cozzolino, Claudia Sarracino, Giuseppina Ratto, Lorena Incorvaia, Nadia Barraco, Giuseppe Badalamenti, Antonio Galvano, Viviana Bazan, Antonio Russo, Giancarlo Troncone, Umberto Malapelle

**Affiliations:** 1 Department of Precision Medicine in Medical, Surgical and Critical Care (Me.Pre.C.C.), University of Palermo, 90127 Palermo, Italy; tancredididier.bazanrusso@unipa.it (T.D.B.R.); valerio.gristina@unipa.it (V.G.); giulia.busuito@you.unipa.it (G.B.); lorena.incorvaia@unipa.it (L.I.); barraconadia@gmail.com (N.B.); giuseppe.badalamenti@unipa.it (G.B.); antonio.galvano@unipa.it (A.G.); antonio.russo@usa.net (A.R.); 2 Division of Medical Oncology, Department of Internal Medicine, The Ohio State University Comprehensive Cancer Center-Arthur G. James Cancer Hospital and Richard J. Solove Research Institute, Columbus, OH 43210, USA; 3 Department of Public Health, University of Naples Federico II, 80131 Naples, Italy; francesco.pepe4@unina.it (F.P.); claudia.scimone@unina.it (C.S.); domenico.cozzolino@unina.it (D.C.); claudia.sarracino@unina.it (C.S.); giuseppina.ratto@unina.it (G.R.); giancarlo.troncone@unina.it (G.T.); 4 Department of Biomedicine, Neuroscience and Advanced Diagnostic (Bi.N.D.), University of Palermo, 90127 Palermo, Italy; viviana.bazan@usa.net

**Keywords:** liquid biopsy, circulating tumor DNA, immune checkpoint inhibitors, non-small-cell lung cancer, molecular residual disease, treatment monitoring, plasma multi-omics, immunotherapy resistance

## Abstract

Immune checkpoint inhibitors (ICIs) have transformed the treatment of non-small-cell lung cancer (NSCLC), yet predicting durable benefit and detecting resistance remain major challenges. As immunotherapy expands across disease stages, minimally invasive tools are increasingly needed to monitor response and inform treatment personalization. Liquid biopsy enables serial assessment of circulating tumor DNA (ctDNA) and other blood-based biomarkers, complementing tissue analysis and imaging. This review critically synthesizes evidence across resectable, locally advanced, and metastatic NSCLC, integrating ctDNA dynamics with emerging multi-omics approaches. Its contribution is to distinguish the prognostic value of circulating biomarkers from evidence that using them to guide treatment improves patient outcomes, while identifying priorities for clinical translation. Across the reviewed studies, ctDNA clearance is associated with favorable outcomes, whereas persistent or rising ctDNA signals an increased risk of recurrence or progression, sometimes before radiological detection. Methylation, fragmentomics, plasma proteomics, and immune-cell profiling may provide complementary information on tumor-immune interactions. However, low tumor shedding, clonal hematopoiesis, assay variability, and unvalidated intervention thresholds limit routine use. The expansion of perioperative immunotherapy and emerging interest in biomarker-guided treatment adaptation make this synthesis timely. Prospective interventional trials are needed to determine whether liquid biopsy can safely guide treatment escalation, de-escalation, or discontinuation. By clarifying both the promise and the limitations of longitudinal monitoring, this review informs research aimed at detecting ineffective treatment earlier and reducing unnecessary treatment exposure while preserving cancer control.

## Simple Summary

Immunotherapy has improved outcomes for people with non-small-cell lung cancer, but some patients do not benefit or develop resistance during treatment. Blood tests known as liquid biopsies may help monitor cancer by detecting tumor-derived DNA and other circulating signals. This review examines their potential across different disease stages, from treatment around surgery to advanced cancer. It also explores how combining these blood-based signals with scans, tumor tissue findings, and clinical information could improve assessment of treatment response and relapse risk. Although promising, these approaches face technical limitations, and earlier detection of molecular changes does not necessarily mean that changing treatment improves outcomes. Further clinical trials are needed before liquid biopsy results can routinely guide treatment intensification, reduction, or discontinuation. The review highlights the evidence and remaining challenges in using these tools to personalize immunotherapy.

## 1. Introduction

Immune checkpoint inhibitors (ICIs) are changing the therapeutic landscape of lung cancer, extending their clinical benefit from advanced disease to resectable and locally advanced stages. The incorporation of anti-PD-L1 antibodies, either alone or in combination with platinum-based chemotherapy or CTLA-4 blockade, has significantly improved survival outcomes across multiple clinical settings, including neoadjuvant, adjuvant, perioperative, and metastatic treatment strategies [1,2]. Nevertheless, durable responses are achieved only in a subset of patients, whereas primary and acquired resistance, together with atypical response patterns such as pseudo-progression and hyper-progression, remain major barriers to precision immunotherapy. These challenges have underscored the need for biomarkers capable not only of predicting treatment benefit but also of dynamically monitoring tumor evolution throughout therapy [3].

Current clinical decision-making relies primarily on tissue-derived biomarkers, particularly PD-L1 expression and tumor mutational burden (TMB). Although these biomarkers have improved patient selection, they are inherently limited by spatial and temporal heterogeneity, sampling bias, and their inability to capture the dynamic interactions between tumor cells and the immune microenvironment. A single tissue biopsy provides only a static snapshot of a continuously evolving disease, failing to reflect clonal evolution, immune editing, emergence of resistance mechanisms, and changes in antigenicity induced by immunotherapy. As ICIs actively reshape tumor biology over time, there is increasing recognition that longitudinal monitoring may be required to complement baseline tissue characterization and optimize therapeutic decision-making [4].

The term “liquid biopsy” encompasses the analysis of multiple analytes contained in biological fluids. Although most commonly associated with peripheral blood, such a tool can dynamically monitor subclonal changes in the tumor molecular profile and longitudinally track molecular drivers across multiple time points [5].

Liquid biopsy has emerged as a promising solution to these limitations by enabling minimally invasive and repeatable assessment of tumor-derived analytes released into the circulation. Beyond circulating tumor DNA (ctDNA), the liquid biopsy ecosystem has rapidly expanded to include circulating tumor cells (CTCs), extracellular vesicles (EVs), cell-free RNA, methylation signatures, fragmentomics, circulating proteins, immune-cell populations, and blood-derived tumor mutational burden (bTMB). These complementary biomarkers provide systemic and dynamic insights into tumor burden, molecular residual disease (MRD), clonal evolution, immune-tumor interactions, and mechanisms of therapeutic resistance. Moreover, serial liquid biopsy may facilitate earlier identification of treatment response, distinguish pseudoprogression from true progression, detect minimal residual disease following curative-intent therapy, and identify patients who may benefit from treatment escalation or de-escalation strategies [5,6,7].

In this review, we discuss the rationale supporting the integration of liquid biopsy into lung cancer immunotherapy, examine the limitations of current tissue-based biomarkers, and summarize the rapidly evolving clinical evidence across early-stage, locally advanced, and advanced non-small-cell lung cancer (NSCLC). Finally, we highlight possible multi-omics approaches including methylation profiling, fragmentomics, proteomics, extracellular vesicles, and artificial intelligence-driven data integration that could reshape liquid biopsy from a mutation detection tool into a comprehensive platform for real-time precision immuno-oncology.

## 2. Biological Rationale for Liquid Biopsy in Immunotherapy

### 2.1. Tumor-Immune Evolution Under Immunotherapy

Despite the remarkable survival benefits achieved with ICIs, only a subset of patients with NSCLC derive durable clinical benefit, while primary and acquired resistance remain major therapeutic challenges, highlighting the need for biomarkers for dynamically monitoring treatment efficacy and tumor evolution. ICIs do not simply induce tumor regression but actively reshape tumor evolution through a process of continuous interaction between the immune system and cancer cells. Under the selective pressure exerted by activated T lymphocytes, highly immunogenic tumor clones are preferentially eliminated, whereas resistant subclones harboring immune-evasive features survive and progressively expand. This dynamic process, known as cancer immunoediting, drives the emergence of acquired resistance and continuously remodels the genomic, transcriptomic, and immunological landscape of the tumor. Consequently, tumor evolution during immunotherapy reflects an ongoing balance between immune-mediated clonal elimination and the selection of resistant populations rather than a static biological state [8,9].

Immune escape can occur through multiple complementary mechanisms acting at different spatial levels. Local mechanisms, including PD-L1 overexpression, impaired antigen presentation, HLA class I loss, and reduced interferon-γ responsiveness, primarily protect individual cancer cells while allowing continued immunoediting of neighboring clones. In contrast, regional mechanisms, such as VEGF-mediated vascular dysfunction, stromal remodeling, and chemokine dysregulation, promote immune exclusion by limiting T-cell trafficking into the tumor microenvironment. Even more profound are global immune-evasion mechanisms, including activation of the WNT/β-catenin pathway, impaired dendritic-cell recruitment, and defective T-cell priming, which broadly suppress antitumor immunity, preserve intratumor heterogeneity (ITH), and ultimately favor resistance to immune checkpoint blockade. Accordingly, increasing ITH has emerged as a hallmark of ineffective immunoediting and has consistently been associated with reduced CD8^+^ T-cell infiltration, diminished cytotoxic activity, inferior responses to PD-1/PD-L1 inhibitors, and poorer clinical outcomes. Moreover, clonal neoantigens shared by all tumor cells generate substantially more effective antitumor immunity than subclonal neoantigens, emphasizing that the quality and evolutionary stability of the antigenic repertoire are at least as important as its quantitative burden [8,10,11].

These evolutionary principles also explain the limitations of currently available tissue biomarkers. Although PD-L1 expression and TMB remain clinically useful, both represent static measurements that incompletely capture the dynamic interaction between tumor evolution and immune selection. High TMB is associated with increased immune-cell infiltration, enhanced antigen presentation, activated interferon signaling, and improved clinical outcomes following PD-1/PD-L1 blockade across PD-L1 expression levels. However, not all mutations contribute equally to durable immunogenicity [12]. These observations reinforce the concept that biomarkers capable of longitudinally tracking clonal evolution, rather than providing a single baseline snapshot, are needed to fully characterize on-treatment tumor-immune interactions.

### 2.2. Limitations of Tissue Biomarkers and the Rationale for Liquid Biopsy

In current clinical practice, biomarkers such as PD-L1 expression and TMB are typically assessed from a single biopsy specimen from a single anatomical site at a single time point, assuming that this sample accurately reflects the entire tumor. However, accumulating evidence indicates that NSCLC exhibits profound spatial and temporal heterogeneity, making this assumption increasingly inadequate for precision immuno-oncology. Tumors continuously evolve under selective pressures imposed by the immune system and systemic therapies, leading to dynamic changes in clonal composition, immune infiltration, antigen presentation, and immune-evasion mechanisms that cannot be captured by a static tissue specimen [13,14].

Among currently available tissue biomarkers, PD-L1 exemplifies these limitations. A comprehensive spatial analysis of 107 resected NSCLCs demonstrated marked ITH, with PD-L1 expression varying substantially across different tumor regions and metastatic sites. Small-scale heterogeneity was observed in 78% of tumors, while nearly half displayed discordant PD-L1 expression between different tissue blocks. Furthermore, 53% of matched primary tumors and lymph-node metastases showed discrepant PD-L1 expression, and approximately one-quarter of patients crossed clinically relevant therapeutic thresholds depending solely on the biopsy location. These findings indicate that a single biopsy may incorrectly classify patients as PD-L1-positive or PD-L1-negative, contributing to the well-recognized observation that some PD-L1-negative patients derive durable benefit from ICIs, whereas many PD-L1-positive patients fail to respond. Importantly, even evaluation of an entire surgical specimen cannot completely overcome this biological heterogeneity, highlighting the intrinsic limitations of tissue-based biomarker assessment [15].

These shortcomings extend beyond baseline patient selection. Tissue biopsies are invasive, often technically challenging, and difficult to repeat serially, particularly in patients with advanced NSCLC or lesions located in anatomically inaccessible sites. Consequently, they provide only a historical snapshot of the disease and are unable to capture real-time tumor evolution during treatment, including clonal selection, emergence of resistance mechanisms, or changes within the tumor immune microenvironment. Liquid biopsy offers a solution to these limitations by enabling minimally invasive, serial assessment of tumor-derived analytes released into the circulation. Tissue samples remain the gold standard for molecular profiling of predictive biomarkers, but spatial heterogeneity and preanalytical issues significantly impact the success rate of molecular profiling in advanced NSCLC patients. LBx samples have proven consistent results in detecting actionable alterations when tissue samples are unavailable for genomic profiling. In detail, LBx has a positive predictive value of 99.0% for identifying molecular alterations in torrent blood, but its analytical negative predictive value spans from 30.0 to 90.0% depending on tumor burden, clinical framework, and heterogeneity in ctDNA shedding [16,17]. Unlike tissue biopsy, circulating biomarkers integrate molecular information from multiple tumor sites and better reflect the systemic and evolving nature of cancer. Serial blood sampling allows continuous monitoring of tumor burden, clonal evolution, treatment response, molecular residual disease, and emerging resistance throughout the disease course. Importantly, dynamic ctDNA kinetics may also complement conventional imaging in distinguishing true progression from atypical response patterns such as pseudo-progression, where radiological enlargement reflects immune-cell infiltration rather than tumor growth. In this setting, declining or undetectable ctDNA despite apparent radiographic progression has consistently been associated with subsequent clinical benefit, suggesting that liquid biopsy may provide an earlier and more biologically meaningful assessment of treatment efficacy than imaging alone [18,19]. Rather than replacing tissue biopsy, liquid biopsy complements conventional pathology by transforming biomarker assessment from a static baseline measurement into a dynamic, real-time evaluation of tumor-immune interactions. This concept provides the biological rationale for incorporating liquid biopsy into immunotherapy, where longitudinal monitoring may ultimately enable more accurate prediction of therapeutic benefit, earlier detection of resistance, and increasingly personalized treatment strategies.

### 2.3. Technical Approaches Measuring ctDNA

Liquid biopsy has become a cornerstone guiding treatment decision-making procedures, dynamically tracking the longitudinal evolution of the molecular profile of tumor patients [20]. Capturing traces of ctDNA across time points, LBx can prevent recurrence early in surgically resected patients or identify high tumor burden in the perioperative setting, selecting patients for adjuvant therapy. In this scenario, ctDNA detection provides a basis for minimal residual disease (MRD) evaluation, supporting dynamic assessment of LBx samples. NGS platforms play a key role in detecting ctDNA in the bloodstream. Heterogeneous NGS strategies are currently available to identify ctDNA traces across clinical points [21,22]. It has been seen that NGS systems investigating MRD can be classified as tumor-informed and tumor-agnostic depending on the need for tumor genotype. In detail, tumor-informed strategies are built to target tumor-private alterations previously detected on paired tissue samples, whereas tumor-agnostic approaches include comprehensive genome profile assays (CGP), mainly WES or WGS, that track tumor signatures. Of note, tumor-informed approaches demonstrated higher analytical sensitivity than agnostic procedures but are affected by scant reference ranges, longer turnaround time (TAT), and consistent technical costs (requiring paired tissue testing). Conversely, tumor-agnostic procedures showed lower sensitivity, TAT, and technical costs than informed strategies [22,23].

## 3. Liquid Biopsy Across Early-Stage and Locally Advanced Lung Cancer Immunotherapy

The expanding role of immunotherapy in early-stage NSCLC has created an increasing need for dynamic biomarkers capable of monitoring treatment response, detecting molecular residual disease (MRD), and refining postoperative risk stratification. To address these challenges, liquid biopsy strategies have evolved from highly personalized tumor-informed assays, which track patient-specific somatic mutations, to tumor-agnostic approaches based on predefined genomic or epigenomic panels that do not require individualized assay design. These different approaches provide distinct advantages, with tumor-informed assays generally offering greater analytical specificity, whereas tumor-agnostic strategies facilitate broader applicability and may improve sensitivity through the integration of genomic and epigenomic biomarkers [24].

### 3.1. Neoadjuvant and Adjuvant Immunotherapy

Across multiple prospective studies in patients receiving neoadjuvant and adjuvant immunotherapy for resectable NSCLC, serial ctDNA assessment consistently provided greater prognostic and predictive value than baseline measurements, highlighting that dynamic molecular response is more informative than pretreatment tumor burden alone. In the phase II LCMC3 trial [25], which enrolled 181 patients treated with two cycles of neoadjuvant atezolizumab, postoperative ctDNA negativity and ctDNA clearance were among the strongest predictors of long-term outcomes, reducing the risk of recurrence and death while complementing major pathological response (MPR) in identifying patients with the most favorable prognosis. Importantly, ctDNA clearance retained prognostic significance even among patients without MPR, reinforcing the concept that pathological response and molecular response capture distinct aspects of treatment efficacy. Similar findings were reported in the multicenter phase II CTONG1804 study [26], in which serial tumor-informed ctDNA monitoring demonstrated progressive molecular clearance throughout treatment, with ctDNA positivity declining from 89.5% at baseline to 34.2% after neoadjuvant therapy and 27.6% after surgery. Patients achieving ctDNA negativity before surgery were substantially more likely to achieve pathological complete response (39.1% vs. 6.7%), while combined ctDNA negativity after neoadjuvant therapy and surgery identified a subgroup with an outstanding 18-month event-free survival of 93.8%, compared with 47.3% among patients with persistent ctDNA.

Beyond ctDNA dynamics, emerging evidence suggests that epigenetic biomarkers may further enhance response prediction. In the prospective RENAISSANCE trial [27], dynamic plasma cfDNA methylation profiling using methylation fragment ratio (MFR) and chromosomal aneuploidy of featured fragments (CAFF) predicted pathological response after only one cycle of neoadjuvant toripalimab plus chemotherapy. Patients demonstrating early biomarker clearance achieved MPR rates exceeding 70%, whereas persistent methylation abnormalities identified poor responders. Furthermore, integration of methylation signatures with PD-L1 expression and immune-cell profiling achieved an AUC of 0.86 for predicting MPR, highlighting the potential of multimodal liquid biopsy approaches that combine genomic, epigenomic, and immune biomarkers.

In the adjuvant setting, the five-year biomarker analysis of the phase III IMpower010 trial [28] demonstrated that postoperative ctDNA positivity identifies patients at highest risk of recurrence following complete resection and platinum-based chemotherapy. Nevertheless, adjuvant atezolizumab improved disease-free survival in both ctDNA-negative and ctDNA-positive patients, indicating that MRD reflects baseline recurrence risk rather than resistance to immunotherapy. Notably, approximately 62% of patients who were ctDNA-positive after surgery achieved molecular clearance following adjuvant chemotherapy, which was associated with substantially improved outcomes, while integration of ctDNA status with PD-L1 expression further refined postoperative prognostic stratification [29].

### 3.2. Perioperative Immunotherapy

**Figure 1:**
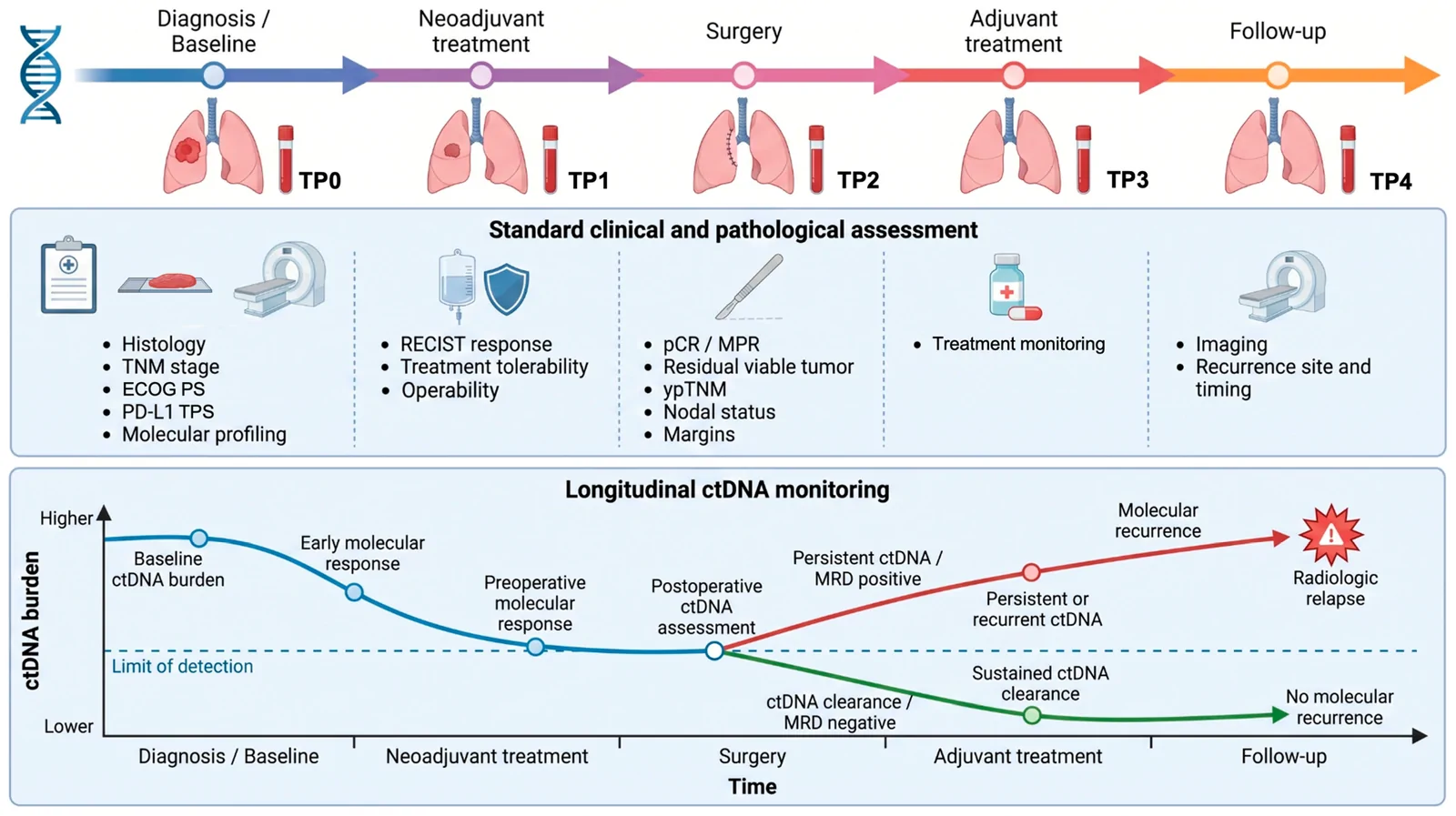
Standard clinicopathological assessment versus longitudinal ctDNA monitoring: complementary approaches to the perioperative management of resectable NSCLC. Abbreviations: ctDNA, circulating tumor DNA; ECOG PS, Eastern Cooperative Oncology Group performance status; MPR, major pathological response; MRD, molecular residual disease; pCR, pathological complete response; PD-L1, programmed death-ligand 1; RECIST, Response Evaluation Criteria in Solid Tumors; TNM, tumor–node–metastasis; TP, time point; TPS, tumor proportion score; ypTNM, post-treatment pathological tumor–node–metastasis classification.

By enabling minimally invasive, longitudinal assessment of ctDNA in patients receiving perioperative immunotherapy, liquid biopsy provides a dynamic measure of tumor response and MRD, striving to complement conventional pathological and radiological evaluation (Figure 1). Prospective studies consistently demonstrate that baseline ctDNA positivity reflects systemic tumor burden, whereas ctDNA clearance during neoadjuvant chemoimmunotherapy is associated with deeper pathological responses and superior long-term outcomes. In a phase II trial including 30 patients with resectable stage II–IIIA squamous NSCLC treated with perioperative serplulimab plus carboplatin and paclitaxel/nab-paclitaxel [30], the regimen achieved an MPR rate of 76.7% and a pathological complete response (pCR) rate of 50%. More than half of the patients (52%) achieved ctDNA clearance before surgery, which predicted pCR with a sensitivity of 78.6%, a specificity of 76.9%, and outperformed both PD-L1 expression and RECIST response (C-index 0.776 vs. 0.667 and 0.655, respectively). Importantly, persistent ctDNA identified occult systemic disease even in a patient achieving pCR who subsequently relapsed, illustrating that pathological eradication does not necessarily equate to molecular cure.

These findings have subsequently been validated in larger randomized perioperative studies. In the phase III trial evaluating perioperative adebrelimab plus platinum-doublet chemotherapy in 501 patients with resectable stage II-III NSCLC [31], immunotherapy significantly improved MPR (53.8% vs. 18.4%), pCR (31.1% vs. 7.6%), and 24-month event-free survival (74.8% vs. 55.5%; HR 0.52). Beyond these clinical benefits, preoperative ctDNA clearance strongly predicted pathological response, with a positive predictive value of 83.1% for MPR and 57.6% for pCR, while persistent postoperative ctDNA identified patients at particularly high risk of recurrence. Similarly, the translational analysis of the phase III CheckMate 77T trial [32] demonstrated that ctDNA clearance before surgery ranked among the strongest predictors of long-term event-free survival, together with definitive surgery and pCR, while baseline ctDNA positivity identified patients deriving substantial benefit from perioperative nivolumab. Extending these observations, the biomarker analysis of the phase III AEGEAN trial [33] demonstrated that failure to clear ctDNA during neoadjuvant durvalumab plus chemotherapy strongly predicted postoperative MRD. Although only 10% of patients remained MRD-positive after surgery, this subgroup experienced dramatically inferior 12-month disease-free survival (14.3% vs. 89.3% in MRD-negative patients), and none achieved either MPR or pCR. Interestingly, exploratory analyses further suggested that Kelch-like ECH-associated protein 1 (*KEAP1*) and Lysine N-methyltransferase 2C (*KMT2C*) alterations were enriched among MRD-positive patients and were associated with reduced benefit from perioperative durvalumab, highlighting the potential value of integrating ctDNA dynamics with tumor genomic profiling for refined risk stratification.

Long-term evidence has been provided by the five-year analysis of the phase II NADIM trial [34], which enrolled 46 patients with resectable stage IIIA NSCLC treated with neoadjuvant nivolumab plus carboplatin and paclitaxel followed by one year of adjuvant nivolumab. Baseline ctDNA burden and, more importantly, ctDNA clearance after neoadjuvant therapy independently predicted progression-free and overall survival. Patients achieving ctDNA clearance experienced outstanding five-year outcomes, with progression-free and overall survival rates of 85.2% and 92.3%, respectively, compared with 60.6% and 59.2% among patients with persistent ctDNA. Interestingly, ctDNA clearance and pCR were only partially concordant, suggesting that pathology primarily reflects local tumor eradication whereas ctDNA captures systemic residual disease. This complementary role was further confirmed in the translational analysis of 60 patients from the randomized NADIM II trial [35], where postoperative MRD assessment using a tumor-agnostic methylation-based assay refined prognostic stratification beyond pCR. Although only 9.6% of postoperative samples were MRD-positive, all MRD-positive patients relapsed, with ctDNA anticipating radiographic recurrence by a median of 7.8 months and identifying a subgroup with an approximately ten-fold higher risk of recurrence and death. Overall, these studies establish serial ctDNA monitoring as one of the most robust dynamic biomarkers in the perioperative setting, supporting its future integration into MRD-guided escalation and de-escalation strategies. However, prospective biomarker-driven interventional trials remain essential before ctDNA-guided therapeutic decisions can be incorporated into routine clinical practice.

### 3.3. Unresectable Stage III Disease

Liquid biopsy is also emerging as a valuable biomarker in unresectable stage III NSCLC, where immunotherapy is administered following or in combination with definitive chemoradiotherapy. Similar to the perioperative setting, serial ctDNA monitoring appears to provide substantially greater prognostic information than baseline measurements, capturing treatment response and molecular residual disease in real time. In the phase II APOLO trial [36], which evaluated induction atezolizumab plus carboplatin–paclitaxel followed by concurrent chemoradiotherapy and maintenance atezolizumab, marked ctDNA reduction during treatment strongly correlated with improved progression-free survival, while baseline ctDNA primarily reflected tumor burden rather than clinical outcome. Dynamic changes in blood tumor mutational burden (bTMB) similarly predicted survival, whereas PD-L1 expression showed limited prognostic value, supporting the concept that longitudinal molecular monitoring may outperform static tissue biomarkers. Complementary evidence comes from the BTCRC LUN 16-081 trial [37], where tumor-informed ctDNA analysis identified molecular residual disease following chemoradiotherapy. Persistent or rising ctDNA after consolidation immunotherapy predicted rapid relapses, with ctDNA anticipating radiographic progression by a median of 4.8 months and identifying patients with an exceptionally poor prognosis. Conversely, ctDNA clearance was associated with durable disease control despite a shortened course of consolidation immunotherapy, providing a rationale for future biomarker-guided escalation and de-escalation strategies.

## 4. Liquid Biopsy in Advanced NSCLC Immunotherapy

In the era of precision oncology, the clinical management of advanced NSCLC has been profoundly reshaped by molecular profiling. Although ICIs have revolutionized the treatment of patients with NSCLC lacking actionable oncogenic drivers, their clinical efficacy remains highly heterogeneous. These limitations have prompted the search for novel biomarkers for patient stratification and guiding treatment decisions.

Several molecular alterations have emerged as potential determinants of response to ICIs. In particular, co-occurring alterations in Kirsten rat sarcoma virus (*KRAS)*, serine/threonine kinase 11 (*STK11*), *KEAP1*, and tumor protein p53 (*TP53*) can substantially affect the prognosis and therapeutic outcomes of patients receiving systemic treatments [38,39,40]. Loss-of-function alterations in *STK11* and *KEAP1* can reshape the tumor microenvironment and tumor metabolism, thereby promoting immune evasion and resistance to ICIs [41]. Accordingly, the efficacy of ICI-based regimens may be markedly influenced by specific driver alterations and their co-mutational context. Nevertheless, clinical trials have generated conflicting evidence regarding the association between individual molecular alterations and outcomes with ICIs.

Several biological factors may partly explain these inconsistent findings. First, co-occurring alterations should be assessed alongside individual driver mutations to capture the broader molecular context. In *KRAS*-mutant lung adenocarcinoma, *STK11/LKB1* co-mutations have been associated with reduced response and shorter survival following PD-1 blockade [42]. *STK11* and *KEAP1* alterations have also been associated with adverse outcomes across different treatment classes, highlighting the need to distinguish their prognostic significance from an ICI-specific predictive effect [43]. The impact of *TP53* co-mutations is similarly context-dependent. In two independent cohorts of ICI-treated lung adenocarcinoma, Scalera et al. observed the shortest survival in *KEAP1*-mutant/*TP53*-wild-type tumors, whereas *KEAP1/TP53* double-mutant tumors exhibited intermediate outcomes, comparable to those of *TP53*-mutant/*KEAP1*-wild-type tumors [44,45]. Second, *STK11* and *KEAP1* alterations constitute a heterogeneous molecular landscape. For example, truncating *STK11* mutations involving exons 1–2 have been associated with a poorer prognosis than alterations involving exons 3–9 [46]. Finally, tumor burden and the clonality of pathogenic alterations have emerged as additional factors that may influence the response to ICIs in patients with NSCLC [47,48].

In this context, alternative strategies for capturing the evolving tumor landscape have become essential. A secondary analysis of the phase III IMpower150 trial showed that patients with *KRAS*-mutated NSCLC harboring plasma co-alterations in *STK11*, *KEAP1*, or *TP53* genes derived greater benefit from ICIs plus chemotherapy than patients with wild-type NSCLC. In this analysis, plasma samples were assessed using the FoundationOne Liquid CDx assay (Foundation Medicine), which interrogates 324 cancer-related genes, including *STK11*, *KEAP1*, and *TP53* [49]. Beyond the detection of individual molecular alterations, complex genomic features have also been investigated in LB samples to improve the stratification of patients with NSCLC who are candidates for ICI-based therapy. Blood-based tumor mutational burden (bTMB) has shown inconsistent associations with clinical outcomes, limiting its routine clinical implementation. Other composite genomic features, including chromosomal instability (CIN) and ITH, have therefore been evaluated as potential biomarkers of ICI sensitivity. However, the low abundance of ctDNA remains a major limitation for the detection of tumor-derived alterations in plasma, particularly in patients with low tumor burden or limited tumor shedding. Broad comprehensive genomic profiling (CGP) assays and highly sensitive analytical approaches may help maximize the information obtained from the limited amount of plasma ctDNA.

CGP has become pivotal for detecting low levels of plasma ctDNA. The phase III CHOICE-01 trial was designed to validate a ctDNA-based model for stratifying patients with NSCLC receiving chemotherapy plus ICIs. In particular, the OncoScreen 520-gene panel (Burning Rock Dx), covering target alterations and complex genomic hallmarks, was selected. A customized bioinformatic pipeline was developed to integrate genomic features and classify patients according to specific molecular patterns, termed blood-based genomic immune subtypes (bGIS), as follows: pretreatment ctDNA-negative (bGIS-1), ICI-favorable (bGIS-2), and ICI-unfavorable (bGIS-3) [50]. CtDNA can also complement conventional tissue-based analyses by tracing molecular drivers across longitudinal samples. In this scenario, the phase II adaptive multi-center BR.36 trial correlated ctDNA levels and radiological response to predict the clinical efficacy of ICIs in NSCLC patients. Interestingly, ctDNA clearance was associated with improved survival when compared with NSCLC patients experiencing MRD [51]. Interestingly, PGDx elio plasma (including *n* = 33 tumor-informed genes) was used to detect low-abundance molecular drivers (MAF 0.25–1.0%) in plasma samples. Longitudinal analysis of clinically relevant time points consistently predicted clinical outcomes of NSCLC patients. Ding et al. evaluated ctDNA status from the Impower 150 trial by adopting an *n* = 311 reference gene hybrid-capture NGS panel (Foundation ACT). The mutation burden detected in plasma decreased significantly after treatment initiation, whereas persistent or increasing ctDNA levels were associated with earlier disease progression and a lower probability of achieving a durable clinical response [52]. It has been established that ctDNA measurement emerges as a prognostic factor for the risk stratification of NSCLC patients, specifically pointing to technical parameters including ctDNA nadir, mutant allele fraction, and ctDNA clearance. Comparing molecular data and radiological response, cfDNA analysis selectively stratified ICI patients by integrating radiological patterns [53]. While targeted liquid biopsy sequencing enables longitudinal tracking of the evolutionary molecular profile and driver landscape, its utility in guiding ICI administration in NSCLC remains constrained by low tumor DNA shedding and the low prevalence of atypical oncogenic drivers. CGP panels, generally covering at least 200 genes, and whole-exome or whole-genome sequencing (WES/WGS) can provide a broader characterization of the tumor molecular landscape. Moreover, CGP assays can incorporate complex genomic metrics through dedicated bioinformatic pipelines. Blood-based TMB, defined as the number of nonsynonymous mutations per megabase of sequenced DNA, has been investigated alongside PD-L1 expression as a potential biomarker of response. In addition, WES analyses have identified recurrent molecular changes at disease progression, including clonal alterations in the *Wnt* pathway, copy-number alterations involving tumor suppressor genes, and remodeling of the tumor microenvironment [54]. Beyond conventional genomic alterations, additional ctDNA-derived features have been investigated as complementary biomarkers. The mutant allele fraction of patient-specific tumor alterations can serve as a surrogate for the circulating tumor fraction and may help predict response to ICI-based therapy (Table 1) [55].

## 5. Beyond Conventional ctDNA Genotyping: Toward a Multi-Omics Liquid Biopsy Ecosystem

### 5.1. Plasma Proteomics

**Figure 2:**
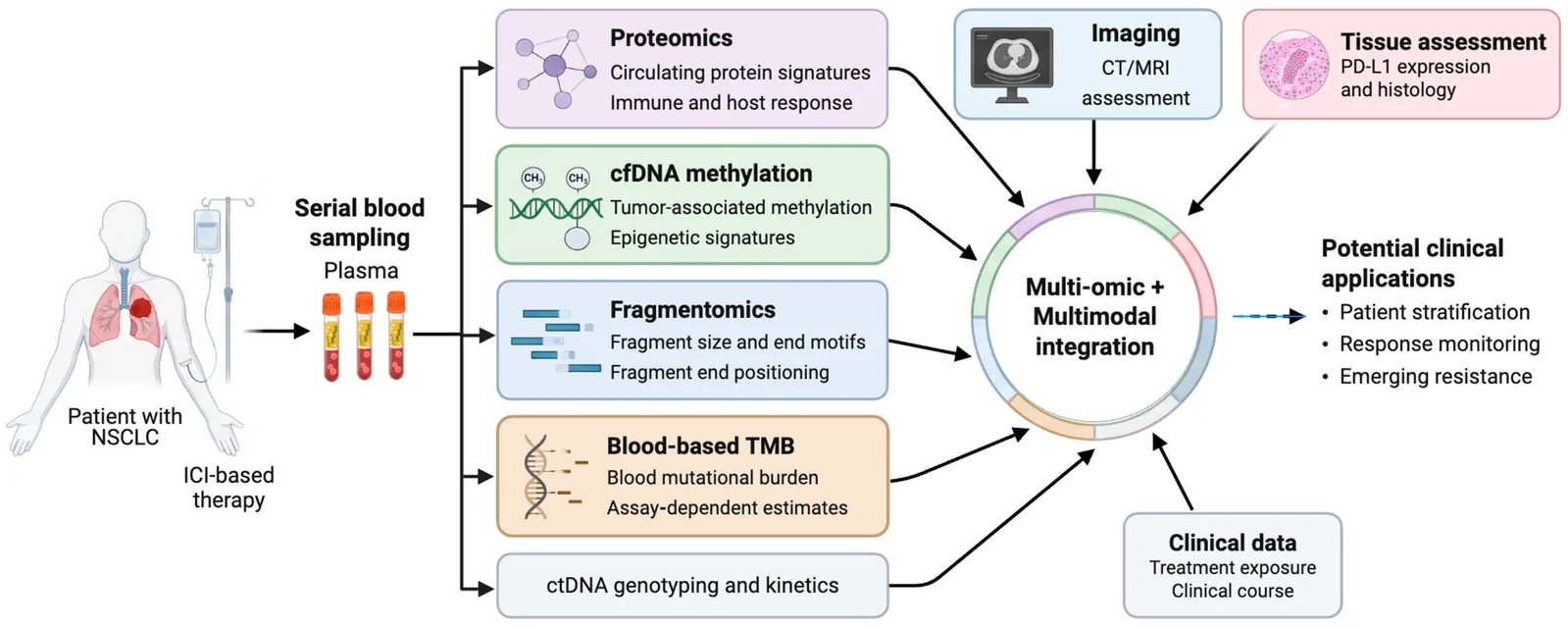
Integration of multi-omic liquid biopsy profiling with imaging, tissue biomarkers, and clinical data in patients with NSCLC receiving immunotherapy. Abbreviations: cfDNA, cell-free DNA; ctDNA, circulating tumor DNA; CT, computed tomography; ICI, immune checkpoint inhibitor; MRI, magnetic resonance imaging; NSCLC, non-small cell lung cancer; PD-L1, programmed death-ligand 1; TMB, tumor mutational burden.

Although ctDNA analysis has become a cornerstone in the clinical management of NSCLC patients, several unmet clinical needs remain. The PD-L1/PD-1 axis plays a key role in selecting patients for ICI therapy. However, the heterogeneous clinical benefit observed within PD-L1-defined populations underscores the need for complementary biomarkers that can improve patient stratification. Beyond multiparametric ctDNA analyses, including genomic, fragmentomic, methylomic, and transcriptomic profiling, plasma proteomics may provide additional information on tumor–host interactions and complement genomic investigations (Figure 2) [57].

Proteomic analysis can capture dynamic changes in the immune system and the host response following treatment-related stimuli. Evidence has shown that elevated CXCL8 and CXCL10 levels are associated with poorer responses to ICIs in patients with NSCLC. However, the comprehensive investigation of proteomic patterns may offer greater predictive value and help stratify patients with NSCLC toward the most appropriate therapeutic option. Aptamers are short DNA or RNA fragments specifically designed to bind selectively to target molecules according to their tertiary structure. The Slow Off-rate Modified Aptamer (SOMA)-based SomaScan assay captures target molecules using aptamers associated with photocleavable linkers and fluorescent labels [58].

A series of 500 patients with NSCLC receiving first-line anti-PD-1/PD-L1 therapy, including 228 and 272 patients in the validation and prospective cohorts, respectively, was profiled for 7596 proteins using the SomaScan Assay v4.1. An interpretive model was developed using 1578 targets that demonstrated the greatest reproducibility across algorithms. Specifically, 388 resistance-associated proteins (RAPs) were identified to stratify patients with first-line ICI-resistant NSCLC. Interestingly, the proteomic score was independent of PD-L1 assessment, thereby identifying two distinct patient populations. Proteomic stratification was associated with clinical outcomes following ICI-based treatment and provided complementary information to PD-L1 expression. In patients with PD-L1 ≥ 50% and a negative PROphet result, chemotherapy plus ICIs was associated with longer overall survival than ICI monotherapy (hazard ratio [HR], 0.23; 95% CI, 0.10–0.51; *p* = 0.0003) [59]. The potential influence of PROphet on treatment selection was evaluated in a study involving 100 oncologists who assessed virtual metastatic NSCLC cases before and after an educational session and disclosure of PROphet results. Treatment recommendations changed in 39.6% of cases after testing. Among cases with PD-L1 ≥ 50%, positive PROphet results increased recommendations for anti-PD-(L)1 monotherapy from 56% to 88%, whereas a negative result increased recommendations for anti-PD-(L)1 plus chemotherapy from 36% to 89%. In cases with PD-L1 < 1% and a negative PROphet result, recommendations for chemotherapy without ICIs increased from 11% to 35%. These findings suggest that proteomic profiling may inform treatment selection alongside PD-L1 expression; however, the virtual-case design did not establish improvements in patient outcomes or reductions in treatment-related toxicity [60]. The minimally invasive nature of liquid biopsy also allows proteomic changes to be monitored longitudinally. Bar et al. analyzed paired pre- and on-treatment plasma samples from 225 patients with NSCLC receiving ICI-based therapy. Overall, 142 proteins showed significantly increased plasma levels following treatment. Soluble PD-1 displayed the greatest increase and, in patients receiving ICI monotherapy, was associated with improved overall survival. Bioinformatic analysis also identified a network of 30 upregulated proteins associated with T-cell activation and recruitment, although this network was detected irrespective of clinical benefit. Conversely, circulating intracellular proteins of alveolar origin were enriched among patients without clinical benefit, potentially reflecting cellular stress and lung tissue injury [61].

Although aptamer-based platforms enable broad proteomic profiling, alternative multiplex technologies, such as proximity extension assays, have been developed to enhance analytical specificity compared with conventional immunoassays. In this scenario, Proximity Extension Assay (PEA™) plays a crucial role in specifically detecting target proteins by updating conventional ELISA assays with massive, parallel, high-throughput sequencing systems. Olink proteomic sequencing assay (Thermo Fisher Scientific), built on DNA oligo-labeled probes, allows for snapshot proteomic assessment, integrating PEA™ with Next-Generation Sequencing (NGS) capturing up to 3.072 proteins in biological samples. A retrospective analysis of *n* = 28 NSCLC patients resistant after anti-*EGFR* treatment was conducted using the Olink Target 96 immune protein panel (Thermo Fisher Scientific). Proteomic analysis highlighted statistically significant variation in terms of durable clinical benefit (DCB) depending on proteomic markers. In particular, IL-4 and IL-6 were significantly higher in DCB, while Gal-9 and GZMH levels correlated with poorer clinical response (median, 1.7 months [95% CI, 1.5–1.9 months) [62]. How immune cells are engaged in PD-L1/PD-1 activation was investigated by adopting the PEA™ system. A total of *n* = 644 proteins were analyzed across longitudinal blood samples, demonstrating that PD-L1 levels increased following ICI administration. Moreover, significant changes in T-cell patterns were observed in patients with NSCLC who experienced clinical benefit. In parallel, subsets of proteins measured at different time points, including baseline and subsequent longitudinal assessments, enabled the clustering of patients with NSCLC and distinguished responders from non-responders [63].

An exploratory analysis on blood samples from *n* = 34 prospective NSCLC patients was carried out to clinically validate the Olink^®^ Immune Oncology Panel (Thermo Fisher Scientific). Interestingly, enrichment analysis selected clinically relevant markers stratifying NSCLC patients on the basis of a prognostic model. In detail, an Immunosuppressive Signature of Combined Resistance Elements (I-SCORE) integrating plasma levels of eight proteins significantly aligned with survival and treatment response [64]. Plasma proteomic profiling effectively monitors treatment resistance following ICI administration, enabling early identification of non-durable clinical benefit. Hierarchical clustering of RAPs aligned with histological subtypes. Integrating RAPs into a multimodal framework alongside clinical and molecular parameters (including PD-L1 expression, genomic alterations, and TMB) yielded strong prognostic accuracy in NSCLC patients [65].

### 5.2. Methylation and Fragmentomics Signatures

Even if PD-L1 evaluation remains a driver guiding first-line ICI-based therapy in advanced NSCLC, predictive performance requires additional investigations. A substantial proportion of patients with high PD-L1 levels (≥50%) fail to achieve durable clinical benefit, whereas long-lasting responses are also observed in PD-L1-negative or low-expressing NSCLC patients. This biological heterogeneity highlights the urgent need for complementary blood-based biomarkers capturing tumor burden and the dynamic interaction of the immune microenvironment. Epigenetic profiling, cfDNA fragmentomics, and bTMB) emerged as promising approaches for stratifying NSCLC patients beyond conventional mutation-based ctDNA analyses [66,67].

DNA methylation represents one of the earliest and most crucial hallmarks reflecting tumor origin and progression. Methylation-based approaches overcome limitations of genomic profiling and comprehensively cover several tumor-specific epigenetic regions, thereby improving analytical sensitivity and enabling longitudinal assessment of tumor dynamics. Hsiao et al. evaluated a methylation-based ctDNA assay [referencing more than 500 cancer-specific hypermethylated genomic loci and calculating a Tumor Methylation Score (TMS)] in a real-world cohort of advanced NSCLC patients treated with anti-PD-1. Interestingly, changes in TMS within 4–10 weeks after treatment initiation showed a remarkable association with real-world progression-free survival (rwPFS) (*p* < 0.0001), whereas the RECIST system demonstrated lower clinical performance during the same interval (*p* = 0.55). Longitudinal TMS correlated with tumor burden, early predicting radiological response or progression by several weeks, when compared to radiological disease re-evaluation [68].

Recent advances in integrating artificial intelligence with cfDNA methylome analysis suggest that circulating epigenetic markers significantly impact the tumor microenvironment. Using a machine-learning algorithm integrating more than 10 million single-cell and spatial transcriptomic profiles across multiple human malignancies, Zhang et al. identified nine highly conserved spatial ecotypes (SEs) consistently aligned with immunotherapy response. In an independent cohort of *n* = 100 melanoma patients, plasma-derived SE levels consistently aligned with immunotherapy outcomes, demonstrating that liquid biopsy can capture evolutionary modification of TM. DNA methylation analysis has emerged as a potentially transformative approach to mapping the spatial immune architecture in lung cancer, serving as a powerful complement to conventional biomarkers for guiding immunotherapy [69].

Beyond epigenetic modifications, fragmentomic analysis recently emerged as a key actor investigating complementary molecular hallmarks affecting tumor patients [70]. Fragmentomics measures fragmentation patterns, including fragment length distribution, end motifs, and fragment end positioning depending on chromatin modifications and/or apoptotic mechanisms. Because these characteristics do not require prior knowledge of tumor-specific genomic alterations, fragmentomic approaches overcome several limitations of personalized ctDNA assays for fingerprinting tumor lesions [71]. Janke et al. [72] demonstrated the feasibility of combining low-coverage whole-genome sequencing (lcWGS) with copy-number variation (CNV) and fragmentomic analyses in *n* = 49 patients with advanced NSCLC receiving PD-L1 blockade. The combined evaluation of CNVs and fragmentation pattern increased ctDNA detection up to 20.3% compared with CNV analysis alone, supporting integrative testing strategies augmenting ctDNA detection rate in biological samples. Notably, baseline fragment length alterations—beyond CNVs—were strongly associated with progression-free survival and significantly improved prediction rate in comparison with PD-L1 expression. Furthermore, persistent CNVs or aberrant fragmentomic profiles during treatment identified a patient subgroup with markedly inferior clinical outcomes, demonstrating a median progression-free survival (PFS) of 5.8 months compared to 47.0 months in those achieving molecular clearance. In multivariable analysis, dynamic ctDNA residual burden—assessed via longitudinal fragmentomics—and PD-L1 expression emerged as the strongest independent predictors of both PFS and overall survival (OS) rates. Consequently, this dynamic plasma-based framework significantly improves prognostic stratification compared to static tissue biomarkers by offering a multiparametric evaluation of circulating cancer signatures.

### 5.3. Blood Tumor Mutational Burden (bTMB)

Blood-based tumor mutational burden (bTMB) has emerged as a promising approach to overcome some of the practical limitations of tissue-based TMB assessment. Compared with tissue-based sequencing, TMB plasma assays are less affected by sampling bias arising from spatial ITH. This concept was first established by Gandara et al. in 2018 in a retrospective analysis of prospectively collected plasma samples from the randomized phase II POPLAR and phase III OAK clinical trials [73,74]. Using a hybrid-capture NGS assay integrating genomic data with TMB measurement, the authors demonstrated that a bTMB cutoff of ≥16 mutations identified previously treated advanced NSCLC patients who experienced greater PFS benefit from atezolizumab compared to docetaxel. Overall findings sustained that bTMB acted as a predictive biomarker for PFS rather than OS, given that in both POPLAR and OAK studies a significant improvement in PFS was achieved for the bTMB ≥ 16 population (HR of 0.57, 95% CI 0.33–0.99 and HR 0.65, 95% CI 0.47–0.92 respectively). Not surprisingly, bTMB also showed partial overlap with PD-L1 expression, suggesting that these biomarkers provide complementary predictive information. Although these findings highlighted the potential application of bTMB, further studies will be warranted to optimize assay design and define clinically meaningful cutoffs. The phase III MYSTIC study [75] was built on the first large-scale validations of bTMB assay using the GuardantOMNI platform. This NGS-based comprehensive technology is a 500-gene panel with a 2.145 Mb sequence output, accounting for SNVs, indels, CNVs, and gene fusions to estimate bTMB. Its bioinformatics workflow removes known germline, driver/resistance, and clonal hematopoiesis-associated variants and incorporates corrections for tumor DNA shedding, enabling a standardized and reproducible assessment of such complex parameters.

Importantly, successful bTMB assessment was achieved in 81.0% of plasma samples compared with only 63.0% of tissue TMB analyses, highlighting the greater feasibility of blood-based testing in routine clinical practice. Cross-validation analyses identified a threshold of ≥20 mutations/Mb as the optimal cutoff for predicting benefit from durvalumab plus tremelimumab versus chemotherapy, while bTMB also showed a significant correlation with tissue TMB (Spearman ρ = 0.60). These results established proof-of-concept that plasma-derived mutational burden can identify patients more likely to benefit from dual immune checkpoint blockade.

Collectively, these observations support that methylation signatures, fragmentomics and bTMB should not be considered competing biomarkers but complementary components of a multidimensional liquid biopsy-based investigation comprehensively analyzing clinically relevant layers beyond relevant tumor–immune interactions, laying the basis for precision immunotherapy on longitudinal multi-omics monitoring (Table 2).

## 6. Current Challenges and Future Directions for Clinical Integration

Despite the expanding evidence supporting liquid biopsy in NSCLC immunotherapy, several analytical, biological, interpretative, and organizational barriers continue to limit its routine clinical integration.

### 6.1. Biological and Analytical Limitations

Assay sensitivity remains closely dependent on tumor shedding, plasma volume, and the number of available template molecules, particularly in patients with low-volume, anatomically confined, or otherwise low-shedding disease. Although error-suppression strategies can detect variants at very low allele frequencies, even highly sensitive platforms cannot recover tumor-derived molecules that are absent from the collected sample; accordingly, a negative result should be interpreted in relation to cfDNA input, assay detection limits, and, whenever possible, the estimated circulating tumor fraction [76]. False-positive findings caused by clonal hematopoiesis represent an additional concern and may require matched leukocyte sequencing or rigorously validated bioinformatic filtering.

### 6.2. Standardization and Clinical Implementation

Variability in blood collection, tube type, processing delay, centrifugation, storage, extraction, sequencing, bioinformatics, and reporting continues to undermine interlaboratory reproducibility. The International Society of Liquid Biopsy has therefore emphasized that clinical reliability requires standardization across the entire pre-analytical, analytical, and post-analytical workflow, together with indication-specific validation, external quality assessment, and transparent reporting of non-informative negative results [77]. Implementation of ISO and CEN/TS standards appears feasible, with one prospective program achieving 96.2% compliance, but required dedicated personnel, training, infrastructure, and continuous sample tracking, illustrating the practical and financial burden of quality-controlled testing [78].

### 6.3. Clinical Interpretation of Molecular Findings

In a retrospective analysis of serial samples from IMpower150, rising ctDNA preceded radiographic progression in 68% of patients with progressive disease and 78% of those with an initially responsive but nondurable course, while longitudinal increases in *STK11*, *KEAP1*, and *SMARCA4* were associated with adverse outcomes [52]. However, molecular progression was not detected in every progressing patient, gene-level analyses were exploratory, and the limited number of atypical responses prevented firm conclusions regarding pseudo-progression or hyper-progression. Liquid biopsy should therefore complement rather than automatically override imaging, pathology, and clinical assessment until prospectively validated intervention thresholds are available. Recent prospective evidence nevertheless demonstrates that implementation may generate practical and economic benefits. In the four-center PLAN study, plasma genotyping was successfully completed in all 100 eligible patients with newly diagnosed advanced non-squamous NSCLC, compared with tissue genotyping in only 68%; plasma results were available 21 days earlier, concordance among paired evaluable samples was 90%, and repeat tissue biopsies were avoided in 12% of patients. Liquid-biopsy genotyping also cost less than half as much as tissue genotyping (€1135 vs. €2404) and produced estimated overall savings of €20,288, partly by preventing inappropriate immunotherapy in patients with sensitizing *EGFR* mutations [79]. However, its single-arm design, country-specific micro-cost model, and omission of fusion detection limit generalizability and reinforce that plasma-first testing should complement, rather than replace, tissue assessment.

### 6.4. Future Directions and Prospective Validation

Future progress will likely depend on moving beyond isolated ctDNA measurements toward integrated models combining ctDNA kinetics with methylation, fragmentomics, plasma proteomics, immune-cell profiling, radiomics, and digital pathology, along with clinical variables evaluation [80]. Artificial intelligence may help integrate these high-dimensional layers and jointly estimate treatment benefit, resistance, and toxicity, but current models remain vulnerable to missing data, batch effects, overfitting, population bias, limited interpretability, and insufficient external validation [81]. Plasma proteomics may also contribute to toxicity monitoring; for example, the IL1RL1/FABP3-based ProIRAE model showed promising discrimination of severe immune-related adverse events, although samples were obtained at toxicity diagnosis, making it a severity-classification tool rather than a validated predictor of future events [82]. Ultimately, the key objective is not simply earlier molecular detection, but evidence that acting on these results improves outcomes. Despite recent advances, ctDNA analysis guiding ICI schemes in perioperative NSCLC patients has not been codified in clinical settings. Biomarker-guided escalation, de-escalation, treatment discontinuation, or intensified toxicity surveillance should therefore be evaluated in prospective, preferably randomized trials with predefined sampling schedules, clinically actionable cut-offs, health-economic analyses, and patient-centered endpoints. In particular, ctDNA clearance is a promising candidate for personalizing ICI duration, but current evidence remains predominantly retrospective and prognostic; until clinical utility is demonstrated, such strategies should remain investigational [83].

## Figures and Tables

**Table 1:** Studies evaluating (**A**) tissue- and (**B**) blood-based biomarkers predicting and monitoring clinical response to immune checkpoint inhibitors (ICIs) in non-small cell lung cancer (NSCLC).

(**A**)					
**Study/** **Authors**	**Setting**	**Biomarkers**	**Study Aim**	**Assay**	**Technical Parameters**
Scalera S. et al. [44]	*n* = 343 tissue samples from ICIs treated advanced/metastatic LUAD.	*KEAP1* and *TP53* mutations.	genomic subtypes predicting immunotherapy outcomes.	FoundationOne CDx (Foundation Medicine, Boston, MA, USA) (324 genes + TMB and MSI); MSK-IMPACT (505 genes); DFCI OncoPanel (447 genes + 60 rearrangement genes).	SE = 2.0–5.0%.
Bange E. et al. [45]	*n* = 44 tissue samples of metastatic/recurrent STK11mt NSCLC.	*STK11*, *KRAS* and *TP53* mutations.	*KRAS* and *TP53* co-mutations predicting outcomes in *STK11*mt patients.	Guardant360^®^ ctDNA assay (Guardant Health, Palo Alto, CA, USA) (74 genes + 18 amplification genes + 7 fusion genes).	SE = 0.1–0.25%.
Pécuchet N. et al. [46]	*n* = 567 resected non-squamous NSCLC.	exon 1–2 and exon 3–9 *STK11* mutations.	prognostic impact of different *STK11* alterations.	Ion AmpliSeq™ Custom DNA Panel (Thermo Fisher Scientific, Waltham, MA, USA) (84 amplicons for 4 genes).	SE = 5.0%.
Frank R. et al. [47]	*n* = 1391 all-stages NSCLC (predominantly advanced/metastatic disease).	*KEAP1* and *NFE2L2* mutations.	clinical/pathological/molecular features predicting clinical outcome in *KEAP1*- and *NFE2L2*-mt cases.	Ion AmpliSeq™ Custom DNA Panel (Thermo Fisher Scientific, Waltham, MA, USA) (12 genes); FISH.	SE = ~5.0%.
IMpower150 trial (phase III)/West HJ. et al. [49]	*n* = 920 for mutational status; *n* = 774 for PD-L1 aNSCLC.	*KRAS*, *STK11*, *KEAP1*, *TP53* mutations; PD-L1 expression.	impact of *KRAS* co-mutations and PD-L1 expression on efficacy of Atezo-based first-line therapy.	FoundationOne^®^ Liquid CDx (Foundation Medicine, Boston, MA, USA), (324 genes + bTMB + MSI); VENTANA PD-L1 (SP263) Assay (Roche, Basel, Switzerland), IHC.	SE = 0.4–1.0%.
KEYNOTE-042 (phase III)/Mok TSK et al. [56]	*n* = 1274 previously untreated locally a/mNSCLC.	PD-L1 valuation (TPS).	Pembro vs. CT as first-line treatment in PD-L1-positive patients.	PD-L1 IHC 22C3 pharmDx assay (Agilent Technologies, Santa Clara, CA, USA).	≥100 viable tumor cells required for evaluation.
(**B**)					
**Study/Authors**	**Setting**	**Biomarker**	**Study Aim**	**Assay**	**Technical Parameters**
Scalera S. et al. [44]	*n* = 304 LBx from ICIs treated advanced/metastatic LUAD.	*KEAP1* and *TP53* mutation.	genomic subtypes predicting ICIs outcomes.	FoundationOne CDx (Foundation Medicine, Boston, MA, USA) (324 genes + TMB and MSI); MSK-IMPACT (505 genes); DFCI OncoPanel (Dana-Faber cancer institute, Boston, MA, USA) (447 genes + 60 rearrangement genes).	SE = 2.0–5.0%.
Bange E. et al. [45]	*n* = 18 LBx of metastatic/recurrent *STK11*mt NSCLC.	*STK11*, *KRAS* and *TP53* mutations.	*KRAS* and *TP53* co-mutations predicting outcomes in *STK11*-mt patients.	Guardant360^®^ ctDNA assay (Guardant Health, Palo Alto, CA, USA) (74 genes + 18 amplification genes + 7 fusion genes).	SE= 0.1–0.25%.
CHOICE-01 (phase III)/Xu J. et al. [50]	*n* = 460 aNSCLC.	bTMB, blood, bITH, bCIN.	ctDNA-based stratification strategy to identify patients benefitting from CT + ICIs.	OncoCompass Plus™ 520-gene panel (Burning Rock Biotech, Guangzhou, Guangdong, China); LP-WGS, ~2× for chromosomal instability.	SE = 0.5%
BR.36 trial (phase II/III)/Anagnostou V. et al. [51]	*n* = 50 aNSCLC.	ctDNA, molecular response (maxMAF clearance).	ctDNA molecular response as early predictor of Pembro efficacy.	TuWBC DNA-informed ultra-deep NGS assay (Personal Genome Diagnostics/Labcorp, Baltimore, MD, USA).	>99.0% for variants at 0.3% MAF and 93.0% at 0.2% MAF.
IMpower150 trial (phase III)/Ding H. et al. [53]	*n* = 466 metastatic NSCLC post-first-line ICIs-therapy.	*STK11*, *KRAS* and *TP53* mutations.	longitudinal ctDNA analysis predicting response/survival in first-line CT + ICIs.	Custom 311-gene hybrid-capture assay (Foundation ACT- Foundation Medicine, Boston, MA, USA).	SE ≥ 0.25–0.5%.
Giroux Leprieur E. et al. [54]	*n* = 8 aNSCLC with durable response and late ICI progression.	Longitudinal ctDNA profiling.	To investigate molecular mechanisms associated with late progression.	Twist Human Core Exome Enrichment System + IntegraGen Custom (Twist Bioscience, South San Francisco, CA, USA).	variants with VAF < 2.0% filtered according to somatic score.

Abbreviations: aNSCLC (advanced NSCLC); bCIN (blood Chromosomal Instability Number); bITH (blood Intratumor Heterogeneity); bTMB (blood Tumor Mutational Burden); ctDNA (circulating Tumor DNA); FISH (Fluorescence In Situ Hybridization); ICIs (Immune Checkpoint Inhibitors); IHC (Immunohistochemistry); LBx (Liquid Biopsy); LP-WGS (Low-Pass Whole-Genome Sequencing); LUAD (Lung Adenocarcinoma); maxMAF (Maximum Mutant Allele Frequency); mt (mutated); *NFE2L2* (*Nuclear Factor*, *Erythroid 2 Like 2*); NGS (Next-Generation Sequencing); NSCLC (Non-Small Cell Lung Cancer); PD-L1 (Programmed Death-Ligand 1); Pembro (Pembrolizumab); PPA (Positive Percent Agreement); *STK11* (*Serine/Threonine Kinase 11*); TMB (Tumor Mutational Burden); *TP53* (*Tumor Protein p53*); TPS (Tumor Proportion Score); TuWBC (Tumor-informed White Blood Cell correction); VAF (Variant Allele Frequency).

**Table 2:** Studies evaluating tissue- and blood-based biomarkers evaluating proteomic signatures in NSCLC patients.

Study/Authors	Setting	Investigated Biomarker	Study Aim	Assay	Technical Parameters
Janke F. et al. [72]	*n* = 49 PD-(L)1 blockade in aNSCLC.	CNV-derived biomarkers (CPA and ctCPA scores) and cfDNA fragmentomic profile.	lcWGS predicting and monitoring response.	LcWGS of plasma cfDNA; Illumina NovaSeq 6000 (Illumina, San Diego, CA, USA).	Fragmentomics + CNV approach increases the analytical sensitivity for +20.3%
PROphet study/Christopoulos P. et al. [59]	*n* = 500 mNSCLC.	Plasma proteomic signature (PROphet score) and CB.	develop and validate a plasma proteomics-based test to guide first-line ICIs treatment selection in mNSCLC.	SomaScan^®^ Assay v4.1 (SomaLogic, Boulder, CO, USA) (~7000 protein targets).	Median LoD: 125 fM or 5.3 pg/mL
Bar J. et al. [61]	*n* = 225 NSCLC receiving ICIs-based regimens (pre and post treatment).	Plasma proteomic.	plasma proteomic changes associated with response/resistance to ICIs in early-stage NSCLC.	SomaScan^®^ Assay v4.1 (SomaLogic, Boulder, CO, USA), (~7000 protein targets).	Median LoD: 125 fM or 5.3 pg/mL
Park MS. et al. [62]	*n* = 28 *EGFR*mt NSCLC treated with ICIs after developing TKI-resistance.	Plasma immune proteins (Galectin-9, Granzyme H, IL-4, IL-6)	Identifying resistance biomarkers.	Olink^®^ Target 96 Immuno-O ncology Panel (PEA) (Olink^®^, Salagatan, Uppsala, Sweden) (92 target proteins).	Target-specific LOD (pg/mL)
J-TAIL study/Mouri A.M et al. [63]	*n* = 51 previously treated aNSCLC receiving Atezo.	Peripheral T-cell subsets (PD-1 + CD8/CD4 T cells, Th7R, CXCR3 + Th7R) and plasma proteins.	peripheral blood immune-cell and plasma proteomic biomarkers as predictors of response.	Helios mass cytometry (CyTOF) for immune profiling + Olink^®^ Target (Olink^®^, Salagatan, Uppsala, Sweden) (644 proteins).	Target-specific LOD (pg/mL)
Gao Y. et al. [64]	*n* = 34 discovery cohort + *n* = 30 validation cohort ICIs treated aNSCLC.	I-SCORE and immune-related plasma protein.	develop/validate I-SCORE for predicting response and survival to PD-1/PD-L1 blockade.	Olink^®^ Target 96 Immuno-Oncology Panel PEA (Olink^®^, Salagatan, Uppsala, Sweden) (92 target proteins).	Target-specific LOD (pg/mL)
PROPHETIC study/Harel M. et al. [65]	*n* = 272 aNSCLC that developed ICIs’ resistance.	RAPs.	Inspecting the biological mechanisms behind resistance.	SomaScan^®^ Assay v4.1 (SomaLogic, Boulder, CO, USA) (~7000 protein targets).	Median LoD: 125 fM or 5.3 pg/mL
Hsiao A et al. [68]	*n* = 20 NSCLC anti-PD1 based therapy monitoring.	TMS from methylated ctDNA.	Evaluating early changes in the ctDNA TMS predicting response to ICIs.	Amplicon-based multiplex cfDNA methylation assay (QCT-NGS) for TMS analysis (>500 hypermethylated loci).	LoQ: TMS = 25.
POPLAR/OAK study (phase II/III)/Gandara DR et al. [74]	*n* = 794 biomarker-evaluable patients with previously treated advanced NSCLC: 211 from POPLAR and 583 from OAK; atezolizumab versus docetaxel	bTMB	Development and validation of a plasma bTMB assay to identify patients deriving progression-free survival benefit from atezolizumab versus docetaxel	Foundation Medicine plasma bTMB assay based on targeted next-generation sequencing of 394 genes (Foundation Medicine, Boston, MA, USA)	SNVs with allele frequency ≥ 0.5%; germline variants and predicted driver mutations excluded. bTMB score cutoff ≥ 16, selected in POPLAR and validated in OAK.
MYSTIC study (phase III)/Si H et al. [75]	*n* = 809 first-line Durva ± Treme or CT for mNSCLC.	bTMB.	bTMB assay validation for first-line Durva + Treme benefit in mNSCLC.	GuardantOMNI^®^ ctDNA assay (500 genes) with the GuardantOMNI bTMB algorithm. (Guardant Health, Palo Alto, CA, USA)	SNV sensitivity: 98.7%; indel sensitivity: 97.2% (vs. WES)

Abbreviations: aNSCLC (advanced NSCLC); Atezo (atezolizumab); bTMB (blood Tumor Mutational Burden); CB (Clinical Benefit); CD8/CD4 T cells (Cluster of Differentiation 8/Cluster of Differentiation 4 T cells); cfDNA (cell-free DNA); CNV (Copy Number Variation); CPA (Copy-number Profile Abnormality); CT (Chemotherapy); ctDNA (circulating Tumor DNA); CXCR3 (C-X-C Motif Chemokine Receptor 3); Durva (durvalumab); ICIs (Immune Checkpoint Inhibitors); IL-4 (Interleukin-4); IL-6 (Interleukin-6); I-SCORE (Immunotherapy Score); lcWGS (low-coverage Whole-Genome Sequencing); LoD (Limit of Detection); LoQ (Limit of Quantification); mNSCLC (metastatic NSCLC); NGS (Next-Generation Sequencing); NSCLC (Non-Small Cell Lung Cancer); PD-(L)1 (Programmed Cell Death Protein 1/Programmed Death-Ligand 1); PD-1 (Programmed Cell Death Protein 1); PEA (Proximity Extension Assay); RAPs (Response-Associated Proteins); SNV (Single Nucleotide Variant); Th7R (T helper 7-like cells); TKI (Tyrosine Kinase Inhibitor); TMS (Tumor Mutation Score); Treme (tremelimumab); WES (Whole-Exome Sequencing).

## Data Availability

No new data were created or analyzed in this study. All data discussed in this review are derived from previously published studies cited in the manuscript.
